# Adenosine metabolic signature in circulating CD4+ T cells predicts remission in rheumatoid arthritis

**DOI:** 10.1136/rmdopen-2023-003858

**Published:** 2024-02-17

**Authors:** Philip M Brown, Amy E Anderson, Najib Naamane, Dennis W Lendrem, Ann W Morgan, John D Isaacs, Arthur G Pratt

**Affiliations:** 1 Translational and Clinical Research Institute, Newcastle University, Newcastle Upon Tyne, UK; 2 National Institute of Health and Care Research (NIHR) Newcastle Biomedical Research Centre, Newcastle Upon Tyne Hospitals NHS Foundation Trust and Newcastle University, Newcastle Upon Tyne, UK; 3 Leeds Institute of Cardiovascular and Metabolic Medicine, University of Leeds, Leeds, UK; 4 NIHR Leeds Biomedical Research Centre and NIHR Leeds Medtech and In Vitro Diagnostics Co-operative, Leeds Teaching Hospitals NHS Trust, Leeds, UK

**Keywords:** methotrexate, rheumatoid arthritis, therapeutics

## Abstract

**Objectives:**

Long-term outcomes in rheumatoid arthritis (RA) depend on early and effective disease control. Methotrexate (MTX) remains the first-line disease modifying therapy, however there are no biomarkers with which to identify those most likely to achieve remission. To address this unmet need we explored metabolic pathways involved in MTX mechanism of action within circulating CD4+T cells in a cohort of treatment naive patients with early RA.

**Methods:**

Purified CD4+T cells were isolated from peripheral blood of 68 patients with early RA commencing MTX. The expression of a range of putative MTX metabolism and mechanism of action targets were explored by flow-cytometry and transcriptional analysis. From these data significant predictors of Disease Activity Score 28-C reactive protein (DAS28-CRP) remission (<2.4 at 6 months) were determined by logistic regression (clinical; flow-cytometry data) and linear modelling (gene expression data).

**Results:**

Low baseline DAS28-CRP was associated with remission at 6 months (p=0.02). Expression of the ectonucleotidase CD39, involved in ATP-ADP conversion during adenosine synthesis, was higher on CD4+CD25 High regulatory T cells at baseline in those achieving remission (molecules of equivalent fluorescence 1264 vs 847; p=0.007). Expression of other adenosine signalling elements in CD4+T cells were also upregulated at baseline in patients achieving remission: *AMPD1* (p<0.001), *ADORA2b* (p=0.039) and *ADORA3* (p=0.047). When combined into a single predictive metric, a combination of these variables outperformed baseline DAS28-CRP in prediction of early remission (area under the curve 0.92 vs 0.67, p=0.001)

**Conclusions:**

Adenosine signalling is important in the achievement of early remission with MTX in RA and biomarkers of adenosine activity may hold utility for the stratification of therapy in early disease.

**WHAT IS ALREADY KNOWN ON THIS TOPIC**

- A proportion of patients with early rheumatoid arthritis achieve effective disease control with methotrexate monotherapy, but there are no reliable pretreatment predictors of response.

**WHAT THIS STUDY ADDS**

- Expression of elements of adenosine activity are upregulated pretreatment in those who achieve remission with methotrexate.
- A composite predictive metric of pretreatment Disease Activity Score 28-C reactive protein, CD39, *AMPD1* and *ADORA2b* in circulating CD4 T cells significantly outperforms clinical predictors for early methotrexate-induced remission.

**HOW THIS STUDY MIGHT AFFECT RESEARCH, PRACTICE OR POLICY**

- These data suggest characterisation of immune cell adenosine signalling activity may assist early therapy stratification in rheumatoid arthritis.

## Introduction

Outcomes for patients with rheumatoid arthritis (RA) have been transformed by early intervention, adoption of treat-to-target management strategies and the development of targeted therapy.^1 2^ There are, however, no effective pretreatment biomarkers to help select the most effective treatment for individual patients. International guidelines recommend the conventional synthetic disease modifying anti-rheumatic drug (csDMARD) methotrexate (MTX) as the first line drug for RA, with additional/alternative therapies deployed if the disease is not adequately controlled.^3 4^ MTX is effective for many, but only 40% of newly diagnosed patients experience a 50% improvement in disease activity measures (American College of Rheumatology-50; ACR50),^5^ the remainder requiring treatment escalation to achieve adequate control and avoid accumulation of joint damage. Furthermore, response to MTX is slow and adverse effects, particularly gastrointestinal intolerance, are common. Optimal management strategies would therefore avoid MTX in those unlikely to respond, and a lack of predictive therapeutic biomarkers for MTX represents a major unmet need in RA management.

There are a number of mechanisms by which MTX may exert its action that could inform predictive biomarker development.^6^ An attractive possibility is its potentiation of adenosine generation via blockade of purine processing and accumulation of adenine moieties, not least because adenosine signalling has been shown to contribute to regulatory T-cell mode of action in murine models, with circumstantial evidence also in humans.^7–10^ Elements in this process include cell surface ectonucleotidases, that cleave ATP and AMP (CD39 and CD73, respectively) into active adenosine and adenosine receptors.^11–13^ A range of other potential mechanistic targets exist including MTX and folate transporters and metabolising enzymes, elements of one-carbon metabolism and histidine metabolism as recently explored in the cancer literature.^6 14^

CD4+T cells play a well-documented role in the dysregulated immune response observed in RA, with a potential role for dysfunctional regulatory T cells (Tregs) as well as atypical effector T cells.^15 16^ To this end we have taken advantage of an inception cohort of patients with newly diagnosed RA starting MTX to profile peripheral blood CD4+T cell protein and gene expression and explore putative biomarkers for MTX response.^9 17–19^ This work forms part of a larger discovery cohort investigating early therapy in RA.

## Methods

### Patients

68 consecutive patients >16 years of age were enrolled for this study from the Northeast Early Arthritis Cohort where they (1) were DMARD and glucocorticoid naïve at the time of enrolment (topical/inhaled glucocorticoids permitted), (2) fulfilled 2010 ACR/EULAR diagnostic criteria for RA and (3) were commenced on oral MTX as a first-line DMARD intervention. Concomitant initiation of hydroxychloroquine and/or an intramuscular glucocorticoid bolus were permitted at the time of enrolment providing this was subsequent to baseline blood draw, but individuals prescribed oral glucocorticoids and/or alternative DMARDs at baseline were excluded. All patients were offered monthly appointments in a nurse-led DMARD escalation clinic over a 6-month follow-up period, where treatment decisions (including additional DMARDs and further intramuscular but not oral steroids) were tailored according to treat-to-target guidelines (NICE-NG100) at the discretion of the supervising rheumatologist in this single-centre observational study. Disease activity was determined by four-component Disease Activity Score (28 swollen/tender joints, patient global health and C reactive protein; DAS28-CRP^20^) and recorded at each hospital visit along with all treatment alterations during the follow-up period. The primary outcome was remission at 6 months (±2 months), defined as DAS28-CRP<2.4^21^ without the need for any systemic glucocorticoid treatment beyond a 4-week window from baseline. Blood samples for research were collected at baseline and 1 month after MTX initiation.

### Flow cytometry

**Figure 1:**
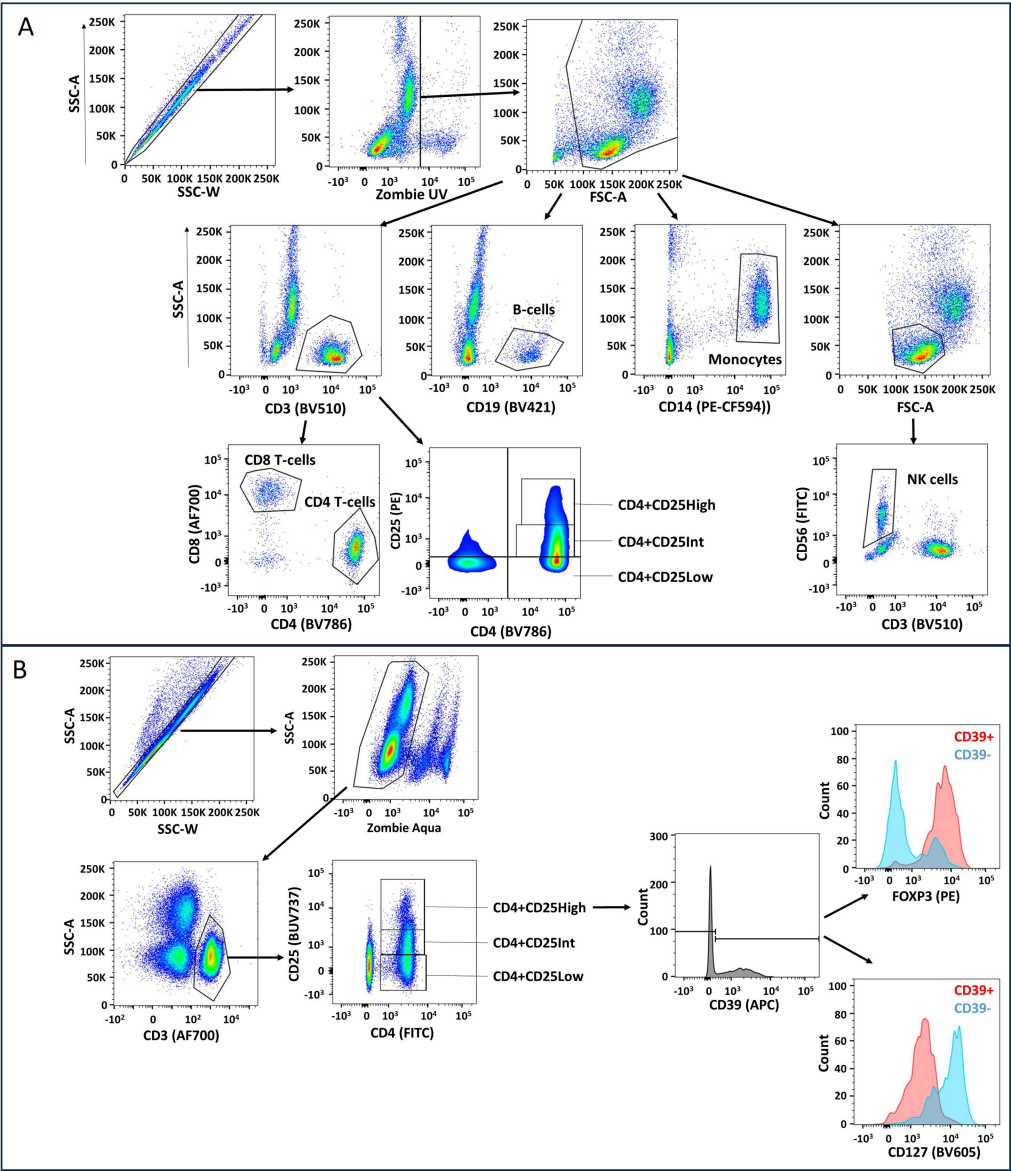
Exemplar gating strategy for flow cytometry data. (A) Cell surface staining strategy with initial singlet and live gates using side scatter and Zombie UV staining followed by exclusion of debris. The resulting ‘Cells’ gate is explored with: CD3 for T cells and subsequent CD8, CD4 and CD25 for CD4+, CD8+ and CD4+CD25 High T cells; CD19 for B cells; CD14 for monocytes and CD56 positive/CD3 negative for NK cells. (B) Intracellular staining strategy with initial singlet and live gates using side scatter and Zombie Aqua followed by T-cell identification with CD3 then CD4 and CD25 High staining to identify the CD25 High expressing CD4+T cells. The FOXP3 and CD127 expression was then explored in the CD39 positive and negative cells. NK, natural killer; APC, allophycocyanin; AF, Alexa-Fluor, B(U)V, brilliant (ultra) violet; FITC, fluorescein isothiocyanate; FSC, forward scatter; SSC, side scatter.

Peripheral blood mononuclear cells (PBMCs) were isolated from blood samples drawn into EDTA by density centrifugation using Lymphoprep (Axis-Shield). Surface protein expression on these PBMCs was determined by flow cytometry to characterise CD39 and CD73 expression using fluorescently labelled antibodies (CD3, CD4, CD8, CD14, CD19, CD25, CD39, CD56, CD73, CD123, CD127, Zombie UV and Zombie Aqua viability dyes—all from Becton, Dickinson and Company (BD) and BioLegend; details in online supplemental table S1). Intracellular staining for FoxP3 was performed using the eBioscience Foxp3/Transcription Factor Staining Buffer Set from Invitrogen. Samples were acquired on a BD LSRFortessa XC20 (BD, Franklin Lakes, USA) with a minimum of 50 000 events captured for each sample. Compensation matrices were created using single stained anti-mouse Ig Compensation Particles (BD) and single stained PBMCs for Zombie UV/Aqua, and molecules of equivalent fluorescence correction performed using 8 peak Rainbow Calibration Particles (BD). The resulting data were analysed using FlowJo software (V.10; BD) with an example of the gating strategy shown in figure 1. Expression of CD39 and CD73 markers was determined by comparison to fluorescence minus one controls to determine the percentage of cells expressing these markers in a given subpopulation; additional biological information was contributed by determining expression as a continuous variable in a population, calculated herein as molecules of equivalent fluorescence (MEF). This involved relating the mean fluorescence intensity (MFI) values for the CD39 and CD73 bound fluorochromes in each sample to stable standards (Sphero 8 peak Rainbow Calibration Particles—BD) run with each sample, thereby converting these MFI values to MEF values, removing noise in the data introduced by the variability in laser performance in the cytometer over time. The potential confounding effects of acquiring samples fresh across a longer time interval were minimised by the recompensation of panel with each acquisition and by performing the MEF correction above to correct for variability in cytometer performance overtime against an internal control of the repeat runs of a batch of calibration beads.

10.1136/rmdopen-2023-003858.supp1 Supplementary data

### Gene expression analysis

A purified CD4+T cell population was derived from the PBMCs using a two-stage magnet assisted cell separation technique. First, monocytes were depleted using CD14 microbeads followed by positive selection of CD4+T cells using CD4 microbeads following manufacturer’s instructions (both from Miltenyi Biotec). Following manufacturer’s instructions RNA was then isolated using Qiagen AllPrep kit before reverse transcription with SuperScript II (Thermo) and subsequent analysis on the BioMark HD microfluidics quantitative PCR system (BioTools). A 16 cycle pre-amplification process was used following a screen of pre-amplification conditions to yield data in the system’s dynamic range for quantification. Drawing on literature sources, we developed a panel of candidate transcripts whose protein products are known to be involved in MTX metabolism and/or that have been previously associated with MTX efficacy as detailed in online supplemental table S2 (TaqMan assays—Thermo Fisher). Gene expression data were extracted using the Fludigm Real-Time PCR analysis software (V.4.7.1) before normalisation and differential expression analysis in R (V.3.6.0) using arrayQualityMetrics (V.3.50.0), HTqPCR (V.1.48.0), sva (V.3.42.0) and limma (V.3.50.0) packages. Delta-CT values were determined relative to the geometric mean of the three most stable of the eight housekeeping genes acquired (namely, *IPO8*, *SDHA* and *POLR2A*).

### Data analysis

Associations between baseline clinical data and remission were determined by forward stepwise logistic regression. Statistically significant clinical predictors (p value<0.05) were included as covariates together with flow cytometry or gene expression data. The differential gene expression analysis was performed using the limma package (V.3.50.0) in the R statistical software (V.3.6.0), by fitting linear mixed-effects models and applying moderated t-tests with a cut-off for unadjusted p values of <0.05 and minimum fold change of 1.5. Adjustments were performed for clinical covariates and for hidden unwanted variation by modelling baseline DAS28-CRP and three surrogate variables, detected using the sva package (V.3.42.0), as fixed effects. To account for the correlation between repeated measurements within patients, technical replication was modelled as a random effect by setting technical replicates as the blocking variable in the duplicateCorrelation and lmFit functions in limma. For the longitudinal data, patient ID was added to the model as a fixed effect to account for the pairing of samples. The false discovery rate was subsequently controlled through a Benjamini-Hochberg multitest correction (MTC). The relative significance of these variables in the final model is expressed using logworth plots of the negative log_10_(p values) and the performance of the model cross-validated using fivefold cross-validation in the JMP Pro software (V.15). The effectiveness of the candidate models predicting DAS28-CRP remission were compared using the DeLong (1988) method for non-parametric comparison of the area under the receiver operating characteristics curves for the models in JMP Pro (V.15).

## Results

### Baseline DAS28-CRP is associated with remission

The baseline clinical and laboratory characteristics of the 68 patients in the cohort are detailed in table 1. These show a typical early RA cohort with median age of 62, and seropositivity rate of approximately 50%. The median follow-up between MTX initiation and treatment response assessment was 5.2 months. All patients received MTX, with 87% receiving a baseline intramuscular steroid. 24% were additionally prescribed hydroxychloroquine from baseline with no other DMARDs prescribed during follow-up. As expected, a lower baseline disease activity score associated with remission (p=0.02), but there were no other significant associations from the clinical data. This included co-prescription of hydroxychloroquine and intramuscular glucocorticoid use at baseline. Based on these findings baseline DAS28-CRP was used as a covariate in subsequent analyses.

**Table 1:** Baseline demographics of recruited cohort by early methotrexate-induced remission status

Number of participants	Remission	Non-remission	P value
	24	44	
Patient age (years)	60 (50–70)	62(51–70)	0.857
Gender (male)	7 (29)	18 (41)	0.486
Active smoker	1 (4)	7 (16)	0.297
Symptom duration (weeks)	12 (8–25)	13 (9–21)	0.802
Early morning stiffness (minutes)	60 (19–180)	60 (30–120)	0.96
Baseline patient global health (0–100)	50 (29–63)	60 (48–76)	0.132
Baseline tender joint count (0–28)	2 (1–5)	4 (1–10)	0.155
Swollen joint count (0–28)	1 (0–3)	4 (1–11)	0.01
Baseline C reactive protein (mg/L)	7 (4–16)	12 (7–27)	0.108
Baseline ESR (mm/hr)	20 (8–31)	25 (9–41)	0.278
Anti-CCP antibody status	15 (62)	22 (50)	0.463
Rheumatoid factor (RF) status	16 (67)	20 (45)	0.155
Double antibody positive (CCP and RF)	15 (62)	18 (41)	0.147
Baseline DAS28-CRP	3.37 (2.93–4.28)	4.69 (3.41–5.26)	0.02
HAQ-DI	1.00 (0.69–1.55)	1.31 (1.03–2.09)	0.177
Baseline steroid given	19 (79)	40 (91)	0.322
Hydroxychloroquine co-therapy	4 (17)	12 (27)	0.493

CCP, Cyclic Citrullinated Peptide; DAS28-CRP, Disease Activity Score 28-C reactive protein; ESR, Erythrocyte Sedimentation Rate; HAQ-DI, Health Assessment Questionnaire - Disability Index.

Baseline clinical, serological and treatment information of the cohort. There was a statistically significant association between lower baseline DAS28-CRP and remission driven principally by a difference in swollen joint count. Data expressed as absolute numbers (percentage of total) and median (IQR).

### Baseline CD39 expression by regulatory CD4+ T cells predicts remission

**Figure 2:**
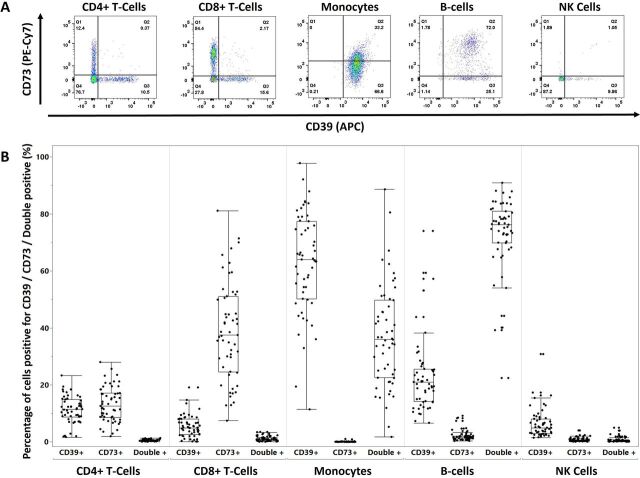
Ectonucleotidase expression on PBMC subsets. (A) Representative flow plots of CD39 and CD73 expression patterns. T cells (CD4+ and CD8+) show single expression of CD39 or CD73 but not both. B cells are predominantly double CD39/CD73 positive. NK cells are predominantly double negative. Monocytes show CD39 expression but variable CD73 expression. (B) Dot and box plots representing baseline CD39 and CD73 expression of indicated PBMC subsets as percentage of cells single positive for CD39 (CD39+), CD73 (CD73+) or co-expressed (double+). APC, allophycocyanin; NK, natural killer; PBMC, peripheral blood mononuclear cell; PE-Cy7, phycoerythrin-cyanine7

**Figure 3:**
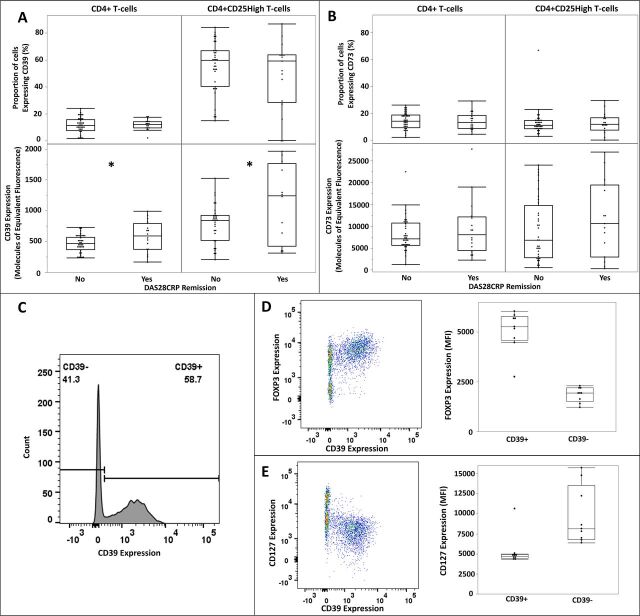
Pretreatment CD39 (A) and CD73 (B) expression on circulating CD4+ and CD4+CD25 High T cells and the FOXP3 and CD127 expression on the CD39+CD4+CD25 High T cells (C, D, E). The expression of CD39 (A) and CD73 (B) are expressed as the percentage of cells positive for the marker determined by fluorescence minus one (FMO) controls (top plots) and as the magnitude of expression expressed as molecules of equivalent fluorescence (bottom plots). (C) Exemplar histogram of CD39 expression in CD4+CD25 High T cells with positive and negative cells gated using FMO. Exemplar pseudocolour plots of FOXP3 (D) and CD127 (E) expression in the CD39+CD4+ CD25 High T cells with boxplots of the medial fluorescence intensity of FOXP3 and CD127 split between CD39+ and CD39− CD4+CD25 High T cells for all samples. n=50 (A, B); n=9 (C, D, E); *p<0.05 (logistic regression using baseline DAS28-CRP as a covariate). DAS28-CRP, Disease Activity Score 28-C reactive protein; MFI, mean fluorescence intensity.

The expression pattern of CD39 and CD73 across CD4, CD8 and B cells, natural killer (NK) cells and monocytes is shown in figure 2. This shows distinct expression patterns, with T cells showing single expression of either CD39 or CD73, B cells showing predominantly co-expression, monocytes showing either CD39 alone or co-expression with CD73 but no CD73 expression alone, and NK cells showing low levels of CD39 alone. Including baseline DAS28-CRP as a covariate, the percentage of CD39 or CD73 expressing CD4 T cells did not differ between patients that subsequently achieved remission versus those who did not (figure 3A,B). In contrast, pretreatment surface expression of CD39 but not CD73 on CD4+T cells was significantly higher among those who subsequently achieved remission (MEF 587 vs 467, p=0.027; figure 3A).

We next examined CD39 and CD73 expression on the Treg subset of CD4+T cells, defined as CD4+CD25 High. This description of Treg cells was selected based on published data and chosen pragmatically due to the absence of routine CD127 on the cell surface staining panel for the samples.^22–24^ As with total CD4+T cells, the percentage of CD39 or CD73 expressing CD4+ T cells did not differ between patients that subsequently achieved remission versus those who did not (figure 3A,B). CD39 but not CD73 expression was again higher among those who subsequently achieved remission compared with those that did not (MEF 1264 vs 847, p=0.007, figure 3A). Intracellular staining of a subset of these samples confirmed the CD39+CD25 hi CD4+T cells to be FoxP3hi, CD127lo Tregs, in contrast to their CD39− counterparts which had heterogeneous expression of both FoxP3 and CD127 (figure 3C–E).

### Differential CD4+ T-cell expression of adenosine pathway components predicts remission

**Figure 4:**
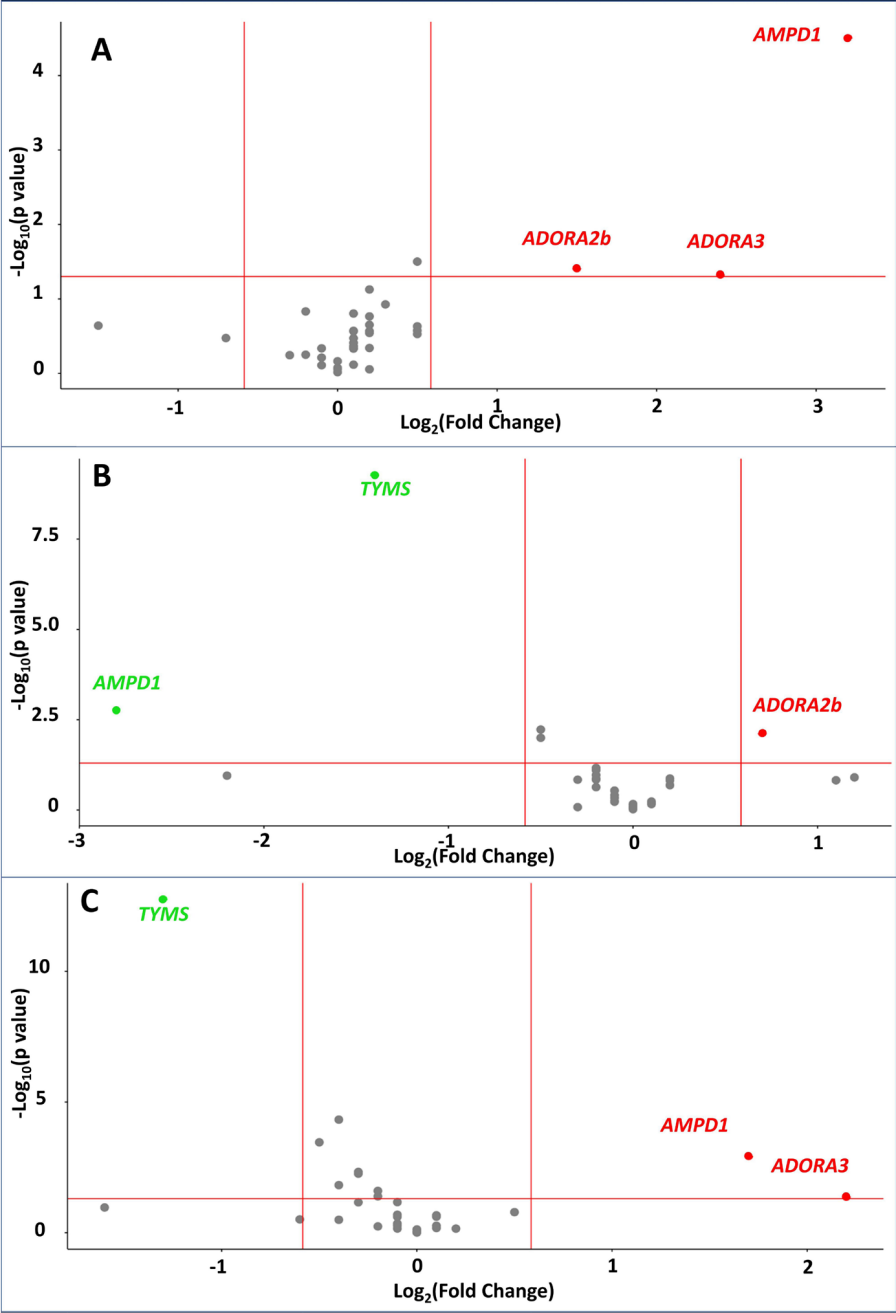
Volcano plots of differentially expressed genes in circulating CD4+T cells. (A) Baseline time point, cross-sectional comparison: gene products significantly upregulated among patients in the early remission compared with non-remission group are depicted in red, and those downregulated in green. (B) Dynamic expression in early remission group: genes significantly upregulated with MTX during the first 4 weeks of treatment are depicted in red, and those downregulated in green. (C) Dynamic expression in non-remission group: genes significantly upregulated with MTX during the first 4 weeks of treatment are depicted in red, and those downregulated in green. The cut-off values for significance were <0.05 (unadjusted) and minimal fold change of >1.5. AMPD1 and TYMS differential expression was significant to multitest correction in all analyses (Benjamini-Hochberg). MTX, methotrexate; TYMS, thymidylate synthetase gene expression.

A volcano plot of pretreatment CD4+T cell expression of the selected candidate genes is shown in figure 4A. Only those involved in adenosine metabolism displayed differential expression between patients with RA who achieved remission and those who did not (p=0.01; hypergeometric test). Most strikingly, AMP deaminase gene expression (*AMPD1*) was 9.4-fold upregulated in those who achieved remission (raw p<0001; MTC p<0.01). The expression of genes encoding two adenosine receptors: *ADORA2b* and *ADORA3* was also higher in remission patients (2.8-fold for *ADORA2b*, raw p=0.039; 5.4-fold for *ADORA3*, raw p=0.047), although these findings were not robust to multiple test correction. The dynamic CD4+T cell expression of candidate genes during the first month of MTX treatment was next considered, in particular seeking longitudinal changes associated with subsequent remission. *AMPD1* expression was observed to be differentially regulated in this way, being *repressed* among patients with RA who progressed to remission at the end of follow-up (0.1-fold expression at 1-month relative to baseline, figure 4B), but 3.3-fold *induced* among those who did not achieve remission (raw p=0.002 and 0.001, respectively; both p<0.05 after multiple test correction, figure 4C). 1.6-fold upregulation of *ADORA2b* expression was also observed in the remission group (raw p=0.007, figure 4B), with 4.5-fold upregulation of *ADORA3* in the non-remission group (raw p=0.042, figure 4C). Finally, downregulation of thymidylate synthetase gene expression following MTX initiation was seen to be common to both outcome groups (figure 4B,C).

### Predictive metric for treatment response

**Figure 5:**
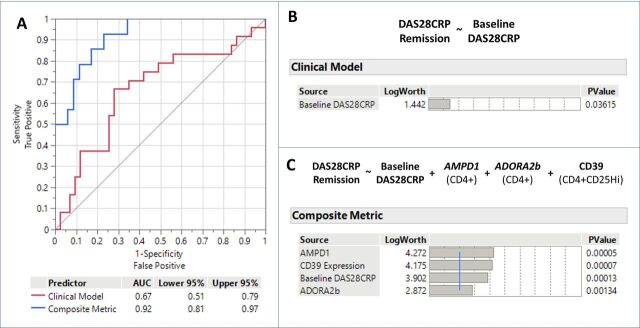
Performance of the clinical and composite predictive metrics for early MTX induced remission outcome in the cohort. (A) Receiver operating characteristics curves comparing the predictive utility of the clinical model (baseline DAS28-CRP only; red) and the composite metric (baseline DAS28-CRP, CD39 expression on CD4+CD25 Hi T cells, baseline AMPD1 and ADORA2b expression in CD4+T cells; blue). The area under the curve (AUC) and 95% CIs are shown with a statistically significant difference in performance between the two models (p=0.001). (B) Logworth plots (−log10(p value)) of the clinical model and (C) logworth plots (−log10(p value)) of the individual components of the composite model showing their significance in the overall model (blue line indicates significance level of 0.01). DAS28-CRP, Disease Activity Score 28-C reactive protein; MTX, methotrexate.

Having identified biomarkers with potential predictive utility for DAS28-CRP remission, a mixed models analysis was undertaken to determine if a composite measure provided more utility than clinical data alone (ie, baseline disease activity). As shown in figure 5A, this indicated that, in combination, CD4+T cell *AMPD1* and *ADORA2b* transcription, baseline disease activity and CD39 expression on CD4+CD25 Hi T cells were significantly associated with DAS28-CRP remission (*ADORA3* expression did not retain statistical significance in the model and was removed). The significance values of the different elements of the model (and baseline DAS28-CRP alone) are shown in figure 5B,C and these associations were robust following fivefold cross-validation. When the model was compared with baseline DAS28-CRP alone for prediction of subsequent DAS28-CRP remission (figure 5A) there was a statistically significant improvement in predictive utility (area under the curve (AUC) combined model 0.922 (95% CI: 0.810 to 0.971) versus AUC baseline DAS28-CRP 0.666 (95% CI: 0.511 to 0.791); p=0.001).

## Discussion

This investigation sought to explore the mechanisms whereby MTX may lead to clinical remission in RA. The data presented strongly implicate a role for adenosine metabolism in remission induction.

The described cohort is broadly similar to that of other early RA cohorts and, as expected, baseline disease activity was a significant predictor of remission^25^; however, no other baseline clinical variables showed a significant association with outcome, including hydroxychloroquine co-therapy.

The cell surface protein adenosine ectonucleotidase CD39, which mediates ATP to AMP cleavage, was more highly expressed at baseline on CD4+T cells of patients achieving remission, particularly on Tregs. The role of CD39 and adenosine in Treg cells has been a focus of interest in relation to tolerance induction. In the mouse, CD25 High CD4 T cells ubiquitously express CD39 and also high levels of CD73.^9^ These cells show immunosuppressive qualities with CD39 knockouts and treatment with adenosine receptor 2a blockade or adenosine deaminase abrogating these immunosuppressive effects. In humans, CD39 expression on CD4+T cells is largely restricted to a Treg population with little CD73 co-expression.^26^ As such, production of adenosine requires interaction with CD73 expressing cells or exosomes.^10^ Given the multiple cell types present in the inflammatory pannus in RA this could be achieved by distinct CD73 expressing CD4+T cells or B cells. Furthermore, CD39 expression and adenosine signalling has been linked to polarisation of naïve T cells to Tregs, with patients expressing higher levels of CD39 showing more potent Treg induction than those with lower expression, with these difference in the low CD39 group being rescued by the addition of CD39 mimetics or ADORA2a agonists. In these studies, the differences in CD39 expression were driven by transforming growth factor-β (TGF-β) signalling via CREB and SMAD.^27^ A similar finding of differential CD39 expression between MTX responders (DAS28<3.0) and non-responders (>4.0) was identified previously, also showing lower adenosine concentrations in the supernatants of ex vivo Treg cultures from non-responders.^11^

Pretreatment bulk CD4+T cell RNA expression identified three transcripts involved in adenosine metabolism that were significantly associated with DAS28CRP remission*—AMPD1*, *ADORA2b* and *ADORA3*. The adenosine receptor most robustly associated with remission from our cohort was ADORA2b. A mouse model of endotoxin lung injury identified a role for ADORA2b signalling in Treg differentiation and an abrogated inflammatory response.^28^ Furthermore, the effect of ADORA2b signalling on dendritic cells is to generate an immune suppressive phenotype, increasing interleukin-10 and TGF-β signalling, suppressing Th1 activity and inducing Treg activity akin to artificially generated tolerogenic dendritic cells.^29 30^ The increased *ADORA2b* expression in MTX-responsive patients in our study may therefore indicate an increased propensity for the induction of Tregs at the site of inflammation on exposure to the drug, and creation of a more immunotolerant environment. Similar expression patterns of ADORA3 and treatment response have been shown from whole blood messenger RNA analysis previously, with higher expression in responders versus non-responders (EULAR response criteria) and upregulation over time with MTX treatment.^31^ The *AMPD1* signal from our cohort shows a differential expression pattern, with enhanced expression in the remission group pretreatment and downregulation with treatment but upregulation with treatment from a lower baseline in the non-responder group. AMPD1 catalyses the conversion of AMP to inosine monophosphate and would therefore be expected to reduce AMP levels available for adenosine generation. AICAR (a purine intermediate compound that accumulates due to MTX action) acts as an AMP mimetic and therefore the AMP status of immune cells may influence their ability to respond to MTX.^32^ Of candidate genes in MTX metabolism, AMPD1 showed the strongest association with disease activity from a recent GWAS.^33^ We also examined expression of several other genes potentially associated with MTX pharmacokinetics and pharmacodynamics (cellular importers and exporters, polyglutamation enzymes, single carbon transfer pathways, histidine metabolism and a range of transcripts previously associated with treatment outcome) and it is remarkable that of these 36 transcripts, only those discussed above were associated with achievement of remission.

In combination the baseline DAS28-CRP, CD4+CD25 High T-cell CD39 expression and CD4+T cell expression of *AMPD1* and *ADORA2b* contribute to a composite predictive ‘signature’, outperforming the clinical variable alone in identifying patients with early RA who subsequently achieve remission with MTX. Remission is, in turn, associated with favourable long-term outcomes, and our findings therefore support a paradigm for biomarker-enhanced treatment decisions in early RA that prioritise more intensive, or alternative, therapeutic intervention for patients in whom timely remission on MTX is unlikely. They furthermore highlight T-cell mediated mechanisms of MTX efficacy—in particular actions of adenosine—that warrant further investigation.

Our data, derived from a prospective study, suggest that those patients achieving the greatest benefit from MTX may do so by being ‘primed’ for an adenosine-driven enhancement of Treg activity. From a clinical perspective the predictive ‘signature’ for DAS28-CRP demands replication in larger validation cohorts, with exploration of more scalable and clinically applicable analysis methods for CD4+T cell transcription such as flow cytometry and/or in situ hybridisation assays. From a mechanistic standpoint the intriguing possibility that, for a subset of patients with early RA, MTX represents a tolerising rather than merely anti-inflammatory therapy, remains to be determined. This is indeed consistent with the clinical observation that a proportion of patients with early RA prescribed MTX can subsequently achieve drug-free remission.^34 35^

## Data Availability

Data are available upon reasonable request. The data underlying this article will be shared on reasonable request to the corresponding author.

## References

[1] van der Linden MPM , le Cessie S , Raza K , et al . Long-term impact of delay in assessment of patients with early arthritis. Arthritis Rheum 2010;62:3537–46. 10.1002/art.27692 20722031

[2] Grigor C , Capell H , Stirling A , et al . Effect of a treatment strategy of tight control for rheumatoid arthritis (the TICORA study): a single-blind randomised controlled trial. Lancet 2004;364:263–9. 10.1016/S0140-6736(04)16676-2 15262104

[3] Smolen JS , Landewé R , Bijlsma J , et al . EULAR recommendations for the management of rheumatoid arthritis with synthetic and biological disease-modifying Antirheumatic drugs: 2016 update. Ann Rheum Dis 2017;76:960–77. 10.1136/annrheumdis-2016-210715 28264816

[4] Fraenkel L , Bathon JM , England BR , et al . American college of rheumatology guideline for the treatment of rheumatoid arthritis. Arthritis Care Res (Hoboken) 2021;73:924–39. 10.1002/acr.24596 34101387 PMC9273041

[5] Hazlewood GS , Barnabe C , Tomlinson G , et al . Methotrexate monotherapy and methotrexate combination therapy with traditional and biologic disease modifying Antirheumatic drugs for rheumatoid arthritis: abridged Cochrane systematic review and network meta-analysis. BMJ 2016;353:i1777. 10.1136/bmj.i1777 27102806 PMC4849170

[6] Brown PM , Pratt AG , Isaacs JD . Mechanism of action of methotrexate in rheumatoid arthritis, and the search for biomarkers. Nat Rev Rheumatol 2016;12:731–42. 10.1038/nrrheum.2016.175 27784891

[7] Haskó G , Cronstein B . Regulation of inflammation by adenosine. Front Immunol 2013;4:85. 10.3389/fimmu.2013.00085 23580000 PMC3619132

[8] Antonioli L , Pacher P , Vizi ES , et al . Cd39 and Cd73 in immunity and inflammation. Trends Mol Med 2013;19:355–67. 10.1016/j.molmed.2013.03.005 23601906 PMC3674206

[9] Deaglio S , Dwyer KM , Gao W , et al . Adenosine generation Catalyzed by Cd39 and Cd73 expressed on regulatory T cells mediates immune suppression. J Exp Med 2007;204:1257–65. 10.1084/jem.20062512 17502665 PMC2118603

[10] Schuler PJ , Saze Z , Hong C-S , et al . Human Cd4+ Cd39+ regulatory T cells produce adenosine upon Co-expression of surface Cd73 or contact with Cd73+ Exosomes or Cd73+ cells. Clin Exp Immunol 2014;177:531–43. 10.1111/cei.12354 24749746 PMC4226604

[11] Peres RS , Liew FY , Talbot J , et al . Low expression of Cd39 on regulatory T cells as a biomarker for resistance to methotrexate therapy in rheumatoid arthritis. Proc Natl Acad Sci U S A 2015;112:2509–14. 10.1073/pnas.1424792112 25675517 PMC4345589

[12] Montesinos MC , Takedachi M , Thompson LF , et al . The antiinflammatory mechanism of methotrexate depends on extracellular conversion of Adenine Nucleotides to adenosine by Ecto-5'-Nucleotidase: findings in a study of Ecto-5'-Nucleotidase gene-deficient mice. Arthritis Rheum 2007;56:1440–5. 10.1002/art.22643 17469101

[13] Montesinos MC , Desai A , Cronstein BN . Suppression of inflammation by low-dose methotrexate is mediated by adenosine A2A receptor but not A3 receptor activation in Thioglycollate-induced Peritonitis. Arthritis Res Ther 2006;8:R53. 10.1186/ar1914 16519795 PMC1526598

[14] Frezza C . Histidine metabolism BOOSTS cancer therapy. Nature 2018;559:484–5. 10.1038/d41586-018-05573-4 30030511 PMC6445353

[15] Cope AP . T cells in rheumatoid arthritis. Arthritis Res Ther 2008;10:S1. 10.1186/ar2412 PMC2582813 19007421

[16] Masoumi M , Alesaeidi S , Khorramdelazad H , et al . Role of T cells in the pathogenesis of rheumatoid arthritis: focus on Immunometabolism dysfunctions. Inflammation 2023;46:88–102. 10.1007/s10753-022-01751-9 36215002

[17] Blits M , Jansen G , Assaraf YG , et al . Methotrexate Normalizes up-regulated folate pathway genes in rheumatoid arthritis. Arthritis Rheum 2013;65:2791–802. 10.1002/art.38094 23897011

[18] Tchetina EV , Demidova NV , Markova GA , et al . Increased baseline Runx2, caspase 3 And P21 gene expressions in the peripheral blood of disease-modifying anti-rheumatic drug-naive rheumatoid arthritis patients are associated with improved clinical response to methotrexate therapy. Int J Rheum Dis 2017;20:1468–80. 10.1111/1756-185X.13131 28741869

[19] Talme T , Bergdahl E , Sundqvist KG . Methotrexate and its therapeutic antagonists caffeine and theophylline, target a Motogenic T-cell mechanism driven by Thrombospondin-1 (TSP-1). Eur J Immunol 2016;46:1279–90. 10.1002/eji.201546122 26909742

[20] Wells G , Becker J-C , Teng J , et al . Validation of the 28-joint disease activity score (Das28) and European League against rheumatism response criteria based on C-reactive protein against disease progression in patients with rheumatoid arthritis, and comparison with the Das28 based on Erythrocyte sedimentation rate. Ann Rheum Dis 2009;68:954–60. 10.1136/ard.2007.084459 18490431 PMC2674547

[21] Fleischmann RM , van der Heijde D , Gardiner PV , et al . Das28-CRP and Das28-ESR cut-offs for high disease activity in rheumatoid arthritis are not interchangeable. RMD Open 2017;3:e000382. 10.1136/rmdopen-2016-000382 28255449 PMC5294021

[22] de Kleer IM , Wedderburn LR , Taams LS , et al . Cd4+Cd25Bright regulatory T cells actively regulate inflammation in the joints of patients with the remitting form of juvenile idiopathic arthritis. J Immunol 2004;172:6435–43. 10.4049/jimmunol.172.10.6435 15128835

[23] Beyer M , Schumak B , Weihrauch MR , et al . In vivo expansion of naive Cd4+ Cd25(High) Foxp3+ regulatory T cells in patients with colorectal carcinoma after IL-2 administration. PLoS One 2012;7:e30422. 10.1371/journal.pone.0030422 22276195 PMC3262821

[24] Bryl E , Daca A , Jóźwik A , et al . Human Cd4Low Cd25High regulatory T cells indiscriminately kill Autologous activated T cells. Immunology 2009;128(1 Suppl):e287–95. 10.1111/j.1365-2567.2008.02961.x 19016909 PMC2753935

[25] RA-MAP Consortium . Characterization of disease course and remission in early Seropositive rheumatoid arthritis: results from the TACERA longitudinal cohort study. Ther Adv Musculoskelet Dis 2021;13:1759720X211043977. 10.1177/1759720X211043977 PMC8544781 34707695

[26] Borsellino G , Kleinewietfeld M , Di Mitri D , et al . Expression of Ectonucleotidase Cd39 by Foxp3+ Treg cells: hydrolysis of extracellular ATP and immune suppression. Blood 2007;110:1225–32. 10.1182/blood-2006-12-064527 17449799

[27] Peres RS , Donate PB , Talbot J , et al . TGF-beta signalling defect is linked to low Cd39 expression on regulatory T cells and methotrexate resistance in rheumatoid arthritis. J Autoimmun 2018;90:49–58. 10.1016/j.jaut.2018.01.004 29426578

[28] Ehrentraut H , Westrich JA , Eltzschig HK , et al . Adora2B adenosine receptor engagement enhances regulatory T cell abundance during Endotoxin-induced pulmonary inflammation. PLoS One 2012;7:e32416. 10.1371/journal.pone.0032416 22389701 PMC3289657

[29] Feoktistov I , Biaggioni I . Role of adenosine A(2B) receptors in inflammation. Adv Pharmacol 2011;61:115–44. 10.1016/B978-0-12-385526-8.00005-9 21586358 PMC3748596

[30] Hilkens CMU , Isaacs JD . Tolerogenic Dendritic cell therapy for rheumatoid arthritis: where are we now Clin Exp Immunol 2013;172:148–57. 10.1111/cei.12038 23574312 PMC3628318

[31] Singh A , Misra R , Aggarwal A . Baseline adenosine receptor mRNA expression in blood as Predictor of response to methotrexate therapy in patients with rheumatoid arthritis. Rheumatol Int 2019;39:1431–8. 10.1007/s00296-019-04344-2 31203399

[32] Baggott JE , Morgan SL , Sams WM , et al . Urinary adenosine and Aminoimidazolecarboxamide excretion in methotrexate-treated patients with psoriasis. Arch Dermatol 1999;135:813–7. 10.1001/archderm.135.7.813 10411156

[33] Taylor JC , Bongartz T , Massey J , et al . Genome-wide Association study of response to methotrexate in early rheumatoid arthritis patients. Pharmacogenomics J 2018;18:528–38. 10.1038/s41397-018-0025-5 29795407

[34] Rayner F , Anderson AE , Baker KF , et al . Biological factors that limit sustAined remission in rhEumatoid arthritis (the BIO-FLARE study): protocol for a non-randomised longitudinal cohort study. BMC Rheumatol 2021;5:22. 10.1186/s41927-021-00194-3 34275488 PMC8286860

[35] Baker KF , Skelton AJ , Lendrem DW , et al . Predicting drug-free remission in rheumatoid arthritis: A prospective Interventional cohort study. J Autoimmun 2019;105:102298. 10.1016/j.jaut.2019.06.009 31280933 PMC6891251

