# Supplementary material for: Adenosine metabolic signature in circulating CD4+ T cells predicts remission in rheumatoid arthritis
Source: RMD Open. 2024 Feb 17;10(1):e003858. doi: 10.1136/rmdopen-2023-003858 (PMC10875551; doi:10.1136/rmdopen-2023-003858)
Supplement: Supplementary data [file rmdopen-2023-003858supp001.pdf]

Supplementary File

A) Surface Stain Flow Cytometry Panel						
**Target**	**Clone**	**Fluorochrome**	**Dilution**	**Spectra**	**Manufacturer**	**Cat #**
CD25	M-A251	PE	1:20	Ex:561 Em:582/15	BD	555432
CD123	7G3	PerCP-Cy5.5	1:20	Ex:488 Em:710/50	BD	558714
CD3	UCHT1	BV510	1:50	Ex:405 Em:525/50	Biolegend	300448
CD4	SK3	BV786	1:50	Ex:405 Em:710/50	BD	563877
CD19	HIB19	BV421	1:100	Ex:405 Em:450/50	Biolegend	302234
CD39	A1	APC	1:100	Ex:635 Em:670/30	Biolegend	328210
CD56	HCD56	FITC	1:50	Ex:488 Em:530/30	Biolegend	318304
CD73	AD2	PE-Cy7	1:50	Ex: 561 Em:780/60	Biolegend	344010
CD8	RPA-T8	AF700	1:50	Ex:635 Em:730/45	BD	557945
CD14	MϕPG	PE-CF594	1:50	Ex:561 Em:610/20	BD	562335
Zombie UV viability dye			1:100	Ex:355 Em:450/50	Biolegend	423108

B) Intracellular FOXP3 Flow Cytometry Panel						
**Target**	**Clone**	**Fluorochrome**	**Dilution**	**Spectra**	**Manufacturer**	**Cat #**
CD3	UCHT1	AF700	1:100	Ex:635 Em:730/45	BD	557943
CD4	RPA-T4	FITC	1:200	Ex:488 Em:530/30	Biolegend	300506
CD25	2A3	BUV737	1:100	Ex:355 Em:730/45	BD	612807
CD39	A1	APC	1:100	Ex:635 Em:670/30	Biolegend	328210
CD73	AD2	PE-Cy7	1:50	Ex: 561 Em:780/60	Biolegend	344010
CD127	A019D5	BV605	1:20	Ex:405 Em:610/20	Biolegend	351334
FoxP3	206D	PE	1:10	Ex:561 Em:582/15	Biolegend	320114
Zombie Aqua viability dye			1:100	Ex:405 Em:525/50	Biolegend	423102

Supplementary Table S1. Flow cytometry panels for A) surface staining of peripheral blood mononuclear cells (PBMCs) and B) intracellular staining for FoxP3. The figures detail the clones, bound fluorophores, dilution of antibodies (determined after in-house titration on PBMCs), excitation and emission spectra and the manufacturer and catalogue number of the used probes. Note, staining for the CD123 was as part of the purity check for the isolated CD4 fraction to identify plasmacytoid dendritic cells. These cells were not abundant enough in the PBMC fraction to be reliably identified and so were not included in the gating strategy.

Target	Protein	Assay ID	Role	Target	Protein	Assay ID	Role	Target	Protein	Assay ID	Role	Target	Protein	Assay ID	Role
RPL14	Ribosomal Protein L14	Hs03004339_g1	Identified from local microarray data of methotrexate survival (continuation of MTX monotherapy for minimum of 4 years)	***SLC19A1***	Solute carrier family member 1	Hs00962908_m1	Cellular importer of MTX	***ADORA1***	Adenosine A1 receptor	Hs00181231_m1	Adenoinse receptors	***18s***	18S Ribosomal RNA	Hs99999901_s1	Housekeeper genes
IGFL2	IGF like family member 2	Hs03645208_g1		***ABCC1***	Multidrug resistance-associated protein 1	Hs01561483_m1	Cellular exporters of MTX	***ADORA2a***	Adenosine A2a receptor	Hs00169123_m1		***RPL13A***	Ribosomal protein L13a	Hs04194366_g1	
PDCD1	Programmed Cell Death 1	Hs01550088_m1		***ABCC2***	Multidrug resistance-associated protein 2	Hs00960489_m1		***ADORA2b***	Adenosine A2b receptor	Hs00386497_m1		***IPO8***	Importin 8	Hs00914057_m1	
LILRB3	Leukocyte Immunoglobulin like receptor B3	Hs01022123_m1		***ABCG2***	ATP binding cassette subfamily G member 2	Hs01053790_m1		***ADORA3***	Adenosine 3 receptor	Hs00181232_m1		***TBP***	TAT-binding protein	Hs00427620_m1	
GRN	Granulin	Hs00963707_g1		***DHFR***	Dihydrofolate reductase	Hs00758822_s1	Target Enzymes of TMX	***ADA***	Adenosine Deaminase	Hs01110945_m1	degredation enzyme of adenosine	***SDHA***	succinate dehydrogenase subunit A	Hs00188166_m1	
GSTM2	glutathione S-transferase mu 2	Hs00265266_g1		***TYMS***	Thymydylate synthase	Hs00426586_m1		***AMPD1***	AMP Deaminase 1	Hs00163633_m1	degredation enzyme of AMP	***POLR2A***	RNA polymerase II subunit A	Hs00172187_m1	
ALOX5	Arachidonate-5-lipoxygenase	Hs00167536_m1		***FPGS***	Folypolyglutamate synthase	Hs00191956_m1	Polyglutamation enzyme of MTX	***ENTPD1***	Ectonucleoside triphosphate dehydrolase-1 (CD39)	Hs00969556_m1	Cleaves extracellular ATP to AMP	***GAPDH***	Glyceraldehyde-3-phosphate dehydrogenase	Hs02786624_g1	
TGFbR1	Transforming growth factor beta receptor 1	Hs00610320_m1	TGFb signalling implicated in Treg expansion and CD39 signalling in RA	***GGH***	Gamma-glutamyl hydrolase	Hs00914163_m1	Deglutamation enzyme of MTX	***NT5E***	5'-ectonucleotidase (CD73)	Hs00159686_m1	Cleaves extracellular AMP to adenosine	***ACTB***	Beta-actin	Hs01060665_g1	
TGFbR2	Transforming growth factor beta receptor 2	Hs00234253_m1		***MTHFR***	Methylene-tetrahydrofolate reductase	Hs01114487_m1	Involved in generation of one-carbon donors from reduced folate	***ATIC***	5-Aminoimidazole-4-Carboxamide Ribonucleotide Formyltransferase/IMP Cyclohydrolase	Hs00269671_m1	Target enzyme of MTX-antagonism results in build up of adenosine moeities				
RUNX2	Runt related transcription factor 2	Hs01047973_m1	Increased baseline expression in peripheral blood associated with better response to MTX	***MAT1A***	Methionine adenosyltransferase 1a	Hs01547962_m1		***HAL***	Histidine ammonia-lysase	Hs00157887_m1	Catalyses first step in histidine metabolism				
CASP3	Caspase 3	Hs00234387_m1		***MTR***	Methionine synthase	Hs01090026_m1		***FTCD***	formiminotransferase cyclodeaminase	Hs01552340_m1	Catalyses folate dependant step in histidine metabolism				
THBS1	Thrombospondin-1	Hs00962908_m1	Possible Thrombospondin-1 related mechanism of response in T-cells	***MTRR***	Methionine synthase reductase	Hs00985015_m1									
				SHMT1	Serine Hydroxymethyltransferase 1	Hs00541043_g1									

Supplementary Table S2. Genes selected for Biomark HD gene expression analysis. The genes were selected based on data implicating a role in therapeutic response prediction from the literature (left hand column) or role in metabolism or mechanism of action of MTX (middle two columns). A range of housekeepers were used (shown in the right-hand column) to allow selection of the most robust for this analysis.

.
